# The Effects of Creatine Monohydrate Supplementation on Recovery from Eccentric Exercise-Induced Muscle Damage: A Double-Blind, Randomized, Placebo-Controlled Trial Considering Sex and Age Differences

**DOI:** 10.3390/nu17111772

**Published:** 2025-05-23

**Authors:** Shota Yamaguchi, Takayuki Inami, Takuya Nishioka, Akihisa Morito, Kaho Ishiyama, Mitsuyoshi Murayama

**Affiliations:** 1 Institute of Physical Education, Keio University, Yokohama 223-8521, Kanagawa, Japan; yamaguchi.s@keio.jp (S.Y.); nishioka.t@keio.jp (T.N.); murayama@keio.jp (M.M.); 2 The Graduate School of Health Management, Keio University, Yokohama 223-8521, Kanagawa, Japan; 3 Taisho Pharmaceutical Co., Ltd., Saitama 331-9530, Saitama, Japan; a-morito@taisho.co.jp (A.M.); ka-ishiyama@taisho.co.jp (K.I.)

**Keywords:** creatine supplementation, exercise-induced muscle damage, muscle recovery, eccentric exercise

## Abstract

**Background/Objectives**: In this study, we aimed to examine the effect of creatine monohydrate (CrM) supplementation on recovery from eccentric exercise-induced muscle damage (EIMD) in diverse populations, including different sexes and age groups. EIMD decreases maximal voluntary contraction (MVC), restricts the range of motion (ROM), and increases muscle stiffness and delayed-onset muscle soreness, all of which negatively impact athletic performance. Therefore, developing effective recovery strategies is essential. **Methods**: A double-blind, randomized, placebo-controlled trial was conducted with 40 healthy male and female participants. After 33 days of supplementation with either CrM or placebo (crystalline cellulose), the participants performed eccentric exercises. Recovery indices, including MVC, muscle stiffness, subjective muscle extensive soreness, fatigue, and upper arm circumference, were measured at baseline, immediately after exercise, 48 h post-exercise, and 96 h post-exercise. **Results**: The creatine supplementation group (CRE) demonstrated a significantly quicker recovery of MVC than the placebo group (PLA). Furthermore, reductions in shear modulus and muscle fatigue were observed in the CRE group. Notably, females in the CRE group exhibited a significant suppression of post-exercise edema, suggesting a sex-specific response. **Conclusions**: These findings indicate that CrM supplementation may enhance recovery from EIMD, contributing to the maintenance of muscle function and the reduction of discomfort after exercise. CrM has the potential to serve as a practical nutritional strategy to promote recovery, not only for athletes, but also for a broader population.

## 1. Introduction

High-intensity eccentric exercise is known to induce exercise-induced muscle damage (EIMD) [1]. EIMD is characterized by muscle cell damage, disruption of excitation–contraction coupling, disorganization of myofibrils, and structural alterations in the extracellular matrix [2]. These physiological changes lead to symptoms such as decreased muscle strength, reduced joint range of motion (ROM), increased muscle stiffness, and delayed-onset muscle soreness [3]. Such symptoms typically emerge within 24 h post-exercise and may persist for several days to a month [3]. In competitive sports, where athletes face consecutive games and training sessions, rapid recovery from EIMD is essential for maintaining performance. However, effective strategies to accelerate recovery from EIMD remain limited, highlighting the need for novel approaches.

Creatine monohydrate (CrM) is widely recognized as an ergogenic aid that supports energy metabolism, promoting muscle hypertrophy and strength gains [4]. Furthermore, CrM facilitates recovery following EIMD. For instance, Yokota et al. [5] demonstrated that CrM supplementation suppresses inflammatory responses after eccentric exercise and alleviates fatigue through its anti-inflammatory effects on muscle and brain tissues. Specifically, the group that consumed CrM exhibited significantly higher spontaneous activity levels post-EIMD and reduced the expression of inflammation-related genes in muscle and brain tissues. These findings suggest that CrM supplementation may accelerate recovery from EIMD and help maintain post-exercise performance [5]. Furthermore, it has been shown that 28 days of CrM supplementation accelerates recovery from EIMD following eccentric exercise. A double-blind, randomized controlled trial involving 20 healthy men divided participants into CrM supplementation and placebo (PLA) groups to evaluate its effects [6]. The results revealed that the CrM group exhibited a significantly higher ROM and maximum voluntary contraction (MVC) than those of the PLA group; furthermore, the arm circumference and shear modulus of the biceps brachii were significantly lower in the CrM group. These findings suggest that CrM supplementation is an effective strategy for accelerating recovery from EIMD [6].

Unfortunately, previous studies have primarily focused on young adult males, leaving the effects of CrM on females and broader age groups insufficiently investigated [7]. Hormonal differences between sexes may influence the degree of EIMD, with estrogen’s anti-inflammatory properties reportedly mitigating EIMD symptoms in females compared to males [8]. Furthermore, aging leads to declines in muscle strength and recovery capacity following EIMD [9], highlighting the importance of examining how CrM supplementation influences these age-related changes. For instance, Lexell [10] reported that aging reduces muscle regeneration capacity, leading to slower recovery following EIMD. Similarly, Faulkner et al. [11] found that structural changes in muscle fibers and increased inflammatory responses associated with aging prolong EIMD recovery. Understanding how these factors interact with the recovery-promoting effects of CrM is crucial for assessing its applicability not only to athletes but also to a wide range of age groups and sexes within the general population. Given the potential influence of hormonal differences and of age-related decline in muscle regeneration and recovery, it is essential to investigate how CrM supplementation interacts with these factors to determine its broader applicability.

The potential mechanisms of action of CrM—such as enhancing cellular membrane stability, attenuating inflammatory responses, and supporting osmotic regulation—may contribute to its recovery-promoting effects. Membrane stabilization prevents calcium influx and subsequent protease activation, which may suppress secondary muscle damage [12,13]. Anti-inflammatory effects may mitigate cytokine-driven soreness and fatigue [5,14], while osmotic regulation may help maintain cell volume and fluid balance [15,16]. Understanding these physiological effects provides a theoretical basis for the potential role of CrM in the improvement of post-exercise recovery across different populations.

In this study, we aimed to investigate whether the previously reported effects of CrM supplementation in males can be replicated in females and individuals across varying age groups. We hypothesized that CrM supplementation would enhance recovery of MVC and ROM, ameliorate muscle stiffness, and reduce soreness and fatigue in both sexes and across age groups. We also expected that female participants might demonstrate greater anti-inflammatory responses to CrM, potentially leading to reduced edema compared to male individuals [8,17]. By elucidating the efficacy of CrM supplementation across different sex and age demographics, this research seeks to advance post-exercise recovery strategies.

## 2. Materials and Methods

### 2.1. Participants

**Figure 1:**
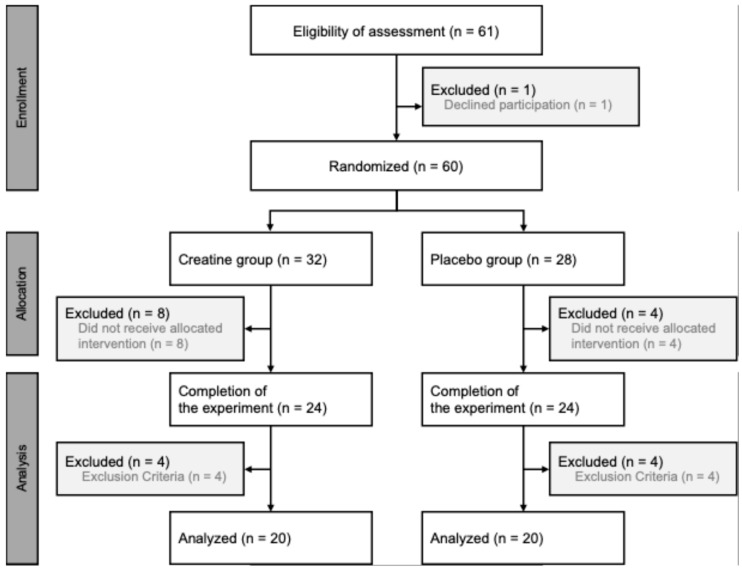
Flowchart illustrating the steps of the parallel randomized controlled trial involving two groups.

This research adhered to the principles of the Declaration of Helsinki (21-003), with written informed consent obtained from all participants after they were thoroughly briefed on the study’s purpose, procedures, and associated risks. An a priori sample size calculation was performed using appropriate input parameters (effect size: 0.2, alpha: 0.05, power: 0.8, correlation: 0.5, ε: 1) for a two-way analysis of variance (ANOVA) (G*Power version 3.1, Heinrich Heine Universität, Düsseldorf, Germany), which suggested a minimum of 36 participants. The study included 40 participants (19 males, 21 females), randomly assigned to either the creatine supplementation (CRE) or PLA group. Randomization was stratified according to the age and maximal muscle strength of the participants to account for inter-individual variability on recovery capacity. Participants ranged from early 20s to mid-40s. The CRE group consisted of 10 males (25.4 ± 7.0 years, MVC: 18.2 ± 2.0 kgf) and 10 females (26.2 ± 7.0 years, MVC: 12.0 ± 1.8 kgf), while the PLA group included 9 males (27.7 ± 9.3 years, MVC: 20.1 ± 3.2 kgf) and 11 females (27.0 ± 8.0 years, MVC: 10.7 ± 1.7 kgf). Conducted at the Keio Physical Education Research Institute in Kanagawa, Japan, from July 2023 to April 2024, the study accounted for the effects of training experience and age on EIMD by randomizing participants based on their maximal muscle strength measurements and age. Randomization was performed using Predictive Analytics Software version 28 for Windows (SPSS Japan Inc., Tokyo, Japan). Based on the selection criteria, 60 participants were enrolled. The inclusion criteria required participants to (1) not be following a weight-loss diet or taking medication, (2) to not have a diagnosis of acute or chronic illness, (3) to not be undergoing any medical treatment that could affect the immune system, (4) to not have any existing injuries, (5) to be non-smokers, (6) to have no history of chronic alcohol abuse, (7) to not consume dietary supplements, and (8) to not have engaged in regular training for more than six months. During the 28-day dietary intake period, 12 participants withdrew for personal reasons. Additionally, among those who completed the study, 8 participants were excluded based on the following criteria: (1) excessive deviation in the residual amount of the test meal, (2) engagement in training during the experimental period, and (3) inability to satisfactorily perform the exercise task. Consequently, a total of 40 participants completed the study and were included in the final analysis (Figure 1).

### 2.2. Experimental Design

This randomized, double-blind, placebo-controlled clinical trial involved participants taking a daily dose of either CrM (3 g each) or microcrystalline cellulose (3 g each), instructed to dissolve the test substance in a meal or water, respectively, before consumption. The CRE and PLA groups consumed CrM and crystalline cellulose (an odorless and tasteless substance), respectively, for a total of 33 days (28 days before and 5 days after the exercise task). Both the CrM and placebo samples were made indistinguishable in appearance. CrM was procured from Nippon Garlic Corporation in Japan (catalog number: creatine-150 m), which sources its creatine monohydrate from AlzChem Trostberg GmbH (Trostberg, Germany), a globally recognized manufacturer of high-purity, pharmaceutical- and food-grade creatine. The product was selected for its verified purity, cost-effectiveness, and established use in previous trials [6]. It was packaged in indistinguishable containers and stored at room temperature to ensure proper blinding throughout the trial. After a 28-day ingestion period, participants performed eccentric exercises. Before the experiment, they attended a familiarization session that included measurements of body composition, MVC, ROM, muscle soreness, muscle fatigue, and arm circumference. On the first day of testing, a pre-exercise warm-up was conducted, followed by eccentric exercises targeting the elbow flexor muscles. The exercises were performed safely as previously described [18]. Measurements of body composition, MVC, ROM, muscle soreness, muscle fatigue, upper-arm circumference, and muscle shear modulus were taken immediately before and after the eccentric exercise routine. These measurements were repeated 48 and 96 h post-exercise. All participants used their left arm for the exercises.

### 2.3. Body Composition

Participants were instructed to wear light athletic clothing and to remove their shoes, as well as any plastic, metal, or easily removable jewelry. Body composition analysis was conducted using a multi-frequency bio impedance analysis (BIA) device (InBody770, InBody Japan Inc., Tokyo, Japan). Participants stepped onto the device, grasped the handrails on both sides, and remained in position for 2 min. The analyzer used an alternating current of 250 mA to assess reactance (Xc), total body water (TBW), extracellular water (ECW), and intracellular water (ICW). Measurements for Xc were obtained at a frequency of 5 kHz to serve as indices of muscle damage [19]. The multi-frequency BIA device measured segmental impedances in the right and left arms, and in the right and left legs at a frequency of 5, 50, and 250 kHz.

### 2.4. Eccentric Exercise

**Figure 2:**
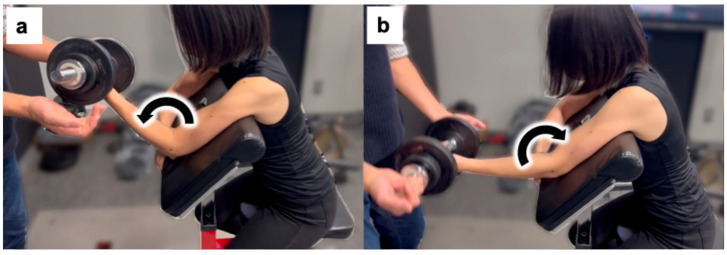
Eccentric exercises involve two phases. In the down (eccentric) phase (**a**), the participant extends their arms in a controlled manner. During the up (concentric) phase (**b**), the examiner lifts the dumbbells. These arrows indicate the movement of the elbow joint. During elbow extension, the participant performed voluntary muscle contraction. In contrast, during elbow flexion, the examiner lifted the dumbbell while the participant maintained a relaxed state.

In this research, the exercise task methodology described [18] was utilized. Participants sat on an arm-curl bench with their hips flexed at an angle of 85° (0° represents full hip extension). They were securely strapped to the bench using non-elastic straps. Participants performed five sets of ten eccentric exercises using dumbbells that weighed 50% of the MVC of their left elbow joint, as recorded during the familiarization session. The elbow joint was extended from 90° to 180° (full extension) at a metronomic pace of 60 beats per min, equating to a 90° extension over 5 s to execute the eccentric actions. The examiner provided support for the elbow flexion during the concentric phase. Each action was repeated every 3 s, with a 2 min recovery period between sets (Figure 2).

### 2.5. Maximum Voluntary Contraction Evaluation

To assess maximal isometric elbow flexion strength, participants performed 5 s MVCs at an elbow angle of 90° using a handheld dynamometer (Mobie; SAKAI Medical Co., Ltd., Tokyo, Japan) [20]. Two trials were conducted, with a third trial added if the difference between the first two measurements exceeded 10%. The highest value recorded was used for evaluation.

### 2.6. Active Range of Motion

A semi-permanent marker was used to mark the center of the acromion, lateral epicondyle, and ulnar styloid. To determine the active ROM of the elbow joint, the joint angle was photographed in both relaxed and flexed states. Using the ImageJ software (version 1.39, U.S. National Institutes of Health, Bethesda, MD, USA) [21], the angle formed between the line connecting the acromion center to the lateral epicondyle and the line connecting the lateral epicondyle to the ulnar styloid was measured. The ROM was calculated by subtracting the angle in the relaxed state from the angle in the flexed state.

### 2.7. Subjective Evaluation

Muscle extensive soreness and fatigue were evaluated subjectively [6]. Both muscle extensive soreness and fatigue were measured using a 100 mm visual analog scale (VAS), where 0 represented no pain (or no fatigue) and 100 represented extreme pain (or extreme fatigue). Muscle extensive soreness was measured during active arm extension by having participants hold their shoulder joints at 90° flexion and their elbow joints at 180° active extension, then marking their perceived soreness levels on the VAS. Muscle fatigue was assessed according to previously reported methods [20].

### 2.8. Circumference

Using a measuring tape (Model R-280; Futaba, Chiba, Japan), the circumference of the upper arm was measured at 50% of the distance from the acromial process of the scapula while the arm hung down by the side. The average of two measurements was recorded [6].

### 2.9. Muscle Shear Modulus

While participants were lying in a supine position with their elbow joint at 180°, shoulder joint extended at 10°, and shoulder joint abducted at 30°, the shear moduli of the biceps brachii muscle (BB) and brachialis muscle (Br) were measured. An ultrasonographic apparatus with an ultrasound shear wave scanner in “shear wave” mode, coupled with a linear array transducer (Aplio 300; Canon Co., Ltd., Tokyo, Japan), was used. The transducer was placed at approximately 50% of the location of the long head of the BB and Br [22]. A semi-permanent ink marker ensured that the probe was consistently placed at the same location across sessions and days, maintaining measurement accuracy throughout the experimental period. Images were captured thrice, once the color map and shear wave speed propagation imaging stabilized for several seconds, during each session. If full elbow extension caused pain, the elbow joint was slowly extended fully while consulting the participant to prevent the stretch reflex [20]. Scanning was performed carefully to avoid muscle compression or deformation. The mean shear modulus was calculated over the largest region of interest, excluding the aponeurosis and subcutaneous adipose tissues from the B-mode images. Elastographic images were saved as bitmap files, measured, and averaged using a built-in software, with the resulting data used for further analyses. All ultrasonographic measurements and data analyses were conducted by an expert with over 20 years of experience. The coefficient of variation for the shear modulus in the resting muscle condition was 2.0% ± 1.9%, with an intraclass correlation coefficient of 0.965 (*p* < 0.001). In this study, the sum of the BB and Br shear moduli was used in the analysis.

### 2.10. Statistical Analysis

The data are presented as means ± standard deviations (SDs). Levene’s test was used to examine equal variances. Baseline values of each variable were compared between the groups using independent *t*-tests. Post-exercise parameter changes between groups (PLA vs. CRE) and between sexes within the CRE group (male vs. female) were analyzed using a two-way ANOVA, considering two factors (group × time). When a significant interaction effect was observed, a post hoc test with Bonferroni’s correction was performed to identify the time points reflecting significant differences between conditions. All raw data and calculated values used for statistical analysis, including measurements at 48 h and 96 h post-exercise, are provided in Table S1. All statistical analyses were conducted using Predictive Analytics Software version 29 for Windows (SPSS Japan Inc., Tokyo, Japan), with statistical significance set at *p* < 0.05.

## 3. Results

### 3.1. Baseline Values

None of the participants who completed the study experienced adverse health effects related to the ingestion of CrM or the placebo. No significant differences were observed in the baseline variables measured prior to the 33-day supplementation period between the PLA and CRE groups (Table 1).

### 3.2. Temporal Changes in Each Muscle Damage Indicator

**Figure 3:**
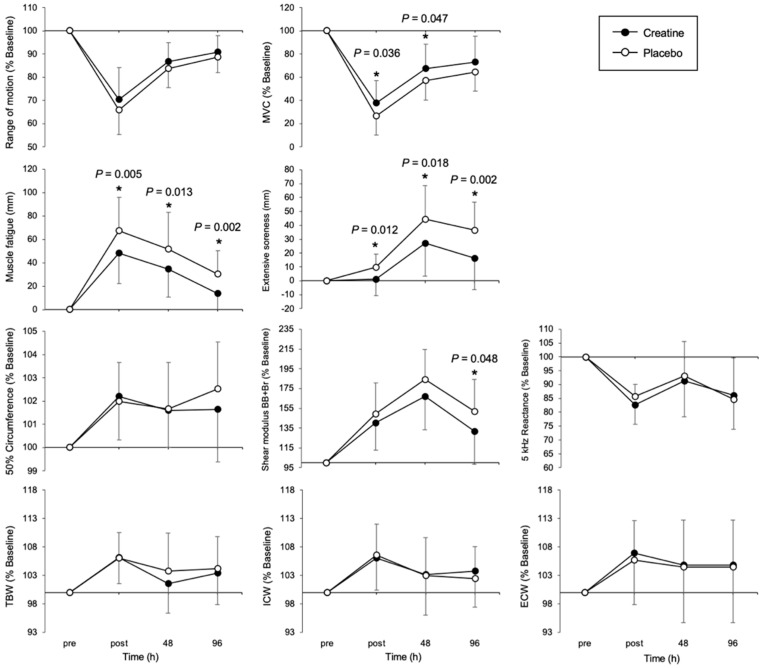
Changes over time in indicators of muscle damage in the creatine (CRE) and placebo (PLA) groups. Temporal changes in MVC, soreness, muscle fatigue, shear modulus, and BIA index between the CRE and PLA groups at the pre-exercise, immediately post-exercise, 48 h, and 96 h time points are shown. MVC, maximum voluntary contraction; BB, biceps brachii; Br, brachialis; TBW, total body water; ICW, intracellular water; ECW, extracellular water; BIA, bio impedance analysis. * *p* < 0.05. Data are presented as means ± SDs.

Comparative data on the physical features of the CRE and PLA groups are presented in Figure 3. A significant between-subjects effect of group was observed for MVC (F (1, 38) = 4.19, *p* = 0.047, partial η^2^ = 0.100), reflecting a moderate effect size. MVC was significantly higher in the CRE group than in the PLA group immediately post-exercise (*p* = 0.036) and at 48 h post-exercise (*p* = 0.047). A significant between-subjects effect of group was observed for muscle fatigue (F (1, 38) = 17.21, *p* < 0.001, partial η^2^ = 0.317), reflecting a moderate effect size. Muscle fatigue was significantly lower in the CRE group immediately (*p* = 0.005), 48 h (*p* = 0.013), and 96 h (*p* = 0.002) post-exercise. A significant between-subjects effect of group was observed for extensive soreness (F (1, 38) = 12.12, *p* = 0.001, partial η^2^ = 0.247), reflecting a moderate effect size. Extensive soreness was significantly lower in the CRE group immediately (*p* = 0.012), 48 h (*p* = 0.018), and 96 h (*p* = 0.002) post-exercise. Furthermore, a significant between-subjects effect of group was observed for shear modulus (F (1, 38) = 3.28, *p* = 0.078, partial η^2^ = 0.080), reflecting a moderate effect size. The shear modulus was significantly lower in the CRE group at 96 h post-exercise (*p* = 0.048).

**Figure 4:**
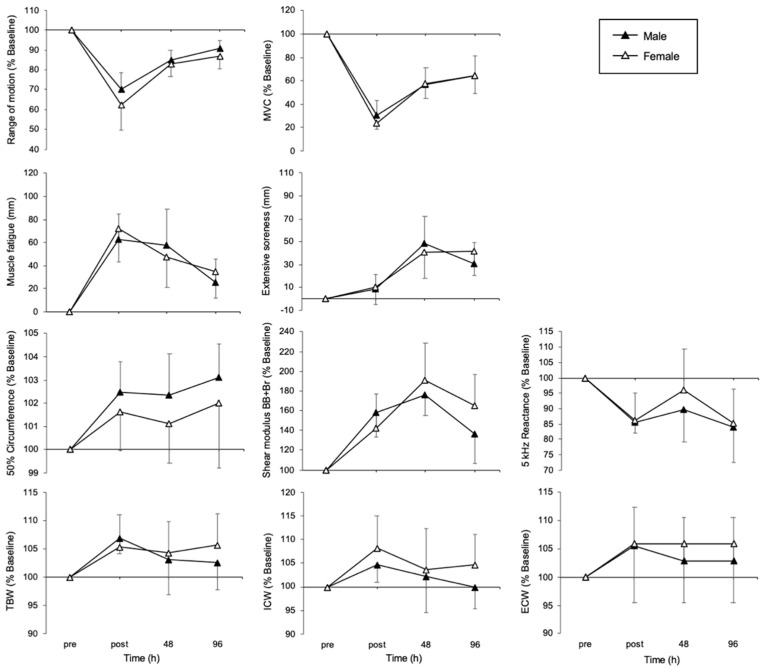
Changes over time in indicators of muscle damage in male and female individuals in the placebo group. Temporal changes in MVC, soreness, muscle fatigue, shear modulus, and BIA index between the male and female groups in the placebo group at the pre-exercise, immediately post-exercise, 48 h, and 96 h time points are shown. MVC, maximum voluntary contraction; BB, biceps brachii; Br, brachialis; TBW, total body water; ICW, intracellular water; ECW, extracellular water; BIA, bio impedance analysis Data are presented as means ± SDs.

**Figure 5:**
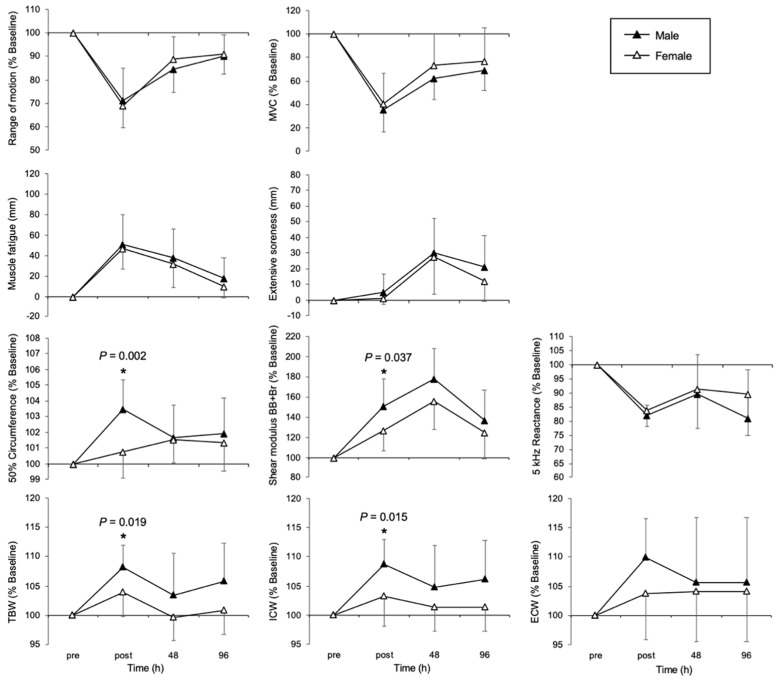
Changes over time in indicators of muscle damage in male and female conditions in the creatine group. Temporal changes in MVC, soreness, muscle fatigue, shear modulus, and BIA index between the male and female groups in the creatine group at pre-exercise, post-exercise, 48 h, and 96 h time points are shown. MVC, maximum voluntary contraction; BB, biceps brachii; Br, brachialis; TBW, total body water; ICW, intracellular water; ECW, extracellular water, BIA, bio impedance analysis * *p* < 0.05. Data are presented as means ± SDs.

Comparative data on physical features by sex in the PLA and CRE groups are shown in Figure 4 and Figure 5, respectively. No significant difference was found for all EIMD indices in the PLA group. Conversely, post-exercise, the circumference at 50% of the limb length, shear modulus, TBW, and ICW were significantly lower among females in the CRE group (circumference: F (1, 38) = 10.48, *p* = 0.002, partial η^2^ = 0.22; shear modulus: F (1, 38) = 4.59, *p* = 0.037, partial η^2^ = 0.11; TBW: F (1, 38) = 5.98, *p* = 0.019, partial η^2^ = 0.14; ICW: F (1, 38) = 6.42, *p* = 0.015, partial η^2^ = 0.14). No significant difference was found for extensive soreness.

## 4. Discussion

In this study, we investigated whether long-term CrM supplementation could promote recovery from EIMD across different age groups and sexes. The results demonstrated that the CRE group exhibited an accelerated recovery of maximum muscle strength, accompanied by a significant suppression of muscle stiffness and fatigue. Furthermore, sex-based analysis revealed that edema was significantly reduced in females compared to males. Previous studies have widely reported the effects of CrM on muscle strength enhancement and athletic performance [23,24], and our previous research [6] demonstrated that CrM supplementation promotes the recovery of maximum muscle strength and ROM following EIMD. In contrast, Doma et al. [25] suggested in a meta-analysis that long-term CrM supplementation might exacerbate muscle damage and delay recovery. However, in this study, long-term CrM supplementation was found to contribute to the promotion of EIMD recovery, regardless of age or sex. The suppression of edema was particularly pronounced in females, a novel finding not previously reported. These results complement existing research by highlighting the broad recovery-promoting effects of CrM across diverse age groups and sexes, supporting the efficacy of long-term supplementation.

This study employed a randomized, double-blind, placebo-controlled design, with a sample size of 40 participants determined through a priori calculations. Randomization was stratified by age and maximum muscle strength to minimize potential confounding factors influencing EIMD. This design aligns with previous research [6] on CrM supplementation and EIMD recovery, enhancing the validity of the protocol. Furthermore, the eccentric exercise protocol used to facilitate EIMD was deemed appropriate, as it successfully elicited sufficient EIMD. For instance, Nosaka et al. [26] reported that eccentric exercise using a dumbbell at 50% MVC reduced the MVC by approximately 50% immediately post-exercise, with recovery approaching approximately 80% after 48 h. In contrast, in this study, MVC in the PLA group decreased to 25% of its maximum value immediately post-exercise and only recovered to approximately 60% after 48 h. These comparisons suggest that the protocol in this study induced stronger muscle damage than that of Nosaka et al. [26], confirming its suitability for evaluating the effects of CrM. Moreover, changes in shear modulus, used as an indicator of muscle stiffness, followed patterns consistent with those observed in previous studies. Specifically, Inami et al. [20] reported that the shear modulus increased approximately 2-fold at 48 h post-exercise and then gradually returned to baseline. In this study, the shear modulus also increased by approximately 1.9-fold, further validating the EIMD induction and recovery process.

In the CRE group, which received CrM supplementation for 33 days, muscle fatigue and the decline in maximum muscle strength were suppressed, while the recovery of muscle stiffness was enhanced. CrM’s contribution to the structural stability of the muscle membrane plays a crucial role in mitigating EIMD. Following EIMD, excessive calcium influx occurs due to membrane disruption, activating proteases such as calpain and caspase [12]. Consequently, the secretion of inflammatory cytokines (interleukin-6 and tumor necrosis factor-alpha) is promoted, leading to secondary inflammation that exacerbates EIMD [13]. In the present study, CrM ingestion was show to contribute in this sequence of processes [14], and it may play an important role, particularly via stabilization of the cell membrane. This stabilization prevents the influx of excess calcium, which in turn prevents the activation of proteases. As a result, the secretion of inflammatory cytokines that trigger secondary inflammation is suppressed and recovery is accelerated. In other words, CrM intake is thought to contribute to the initial stabilization of the cell membrane, which leads to suppression of secondary inflammation and thus accelerates recovery of muscle function. These findings align with those from Santos et al. [27], who reported that CrM supplementation suppresses the rise in serum creatine kinase and lactate dehydrogenase following EIMD. Similarly, in this study, MVC recovery was significantly enhanced in the CRE group compared with the PLA group, suggesting that CrM not only serves as an energy source but also stabilizes cell membranes, reduces secondary inflammation, and facilitates the recovery of muscle function. Overall, these results suggest that CrM may maintain cellular membrane homeostasis after EIMD by suppressing abnormal calcium dynamics, reducing protease activity, and mitigating oxidative stress. Further investigation into detailed mechanisms is warranted.

In this study, 33 days of CrM supplementation suppressed the occurrence of inflammation and edema following eccentric exercise, with a particularly notable suppressive effect observed immediately after exercise in female participants (Figure 5). However, no clear differences between the groups were observed at 48 and 96 h post-exercise. These results suggest that in addition to the stabilizing effects of CrM on cell membranes and its antioxidant properties, the female-specific hormonal environment (in particular, interactions with estrogen) may have alleviated the inflammatory response and increased vascular permeability immediately after exercise. Edema immediately after eccentric exercise is believed to be caused by increased vascular permeability and damage to the muscle cell membrane, which leads to the movement of extracellular fluid into the intermuscular space [28]. CrM has previously been reported to stabilize muscle cell membranes and to play a role in osmotic regulation [15], particularly by maintaining the function of the Na^+^/K^+^ pump, thus helping to maintain the water balance inside and outside of cells [16]. Additionally, CrM is known to have antioxidant effects, and by suppressing excessive production of reactive oxygen species (ROS), it may prevent increased vascular permeability [29]. The suppression of post-exercise edema observed in this study is likely the result of all these mechanisms acting together.

What is particularly interesting is that this effect was more pronounced in females. Estrogen is known to have anti-inflammatory properties, suppressing the infiltration of monocytes/macrophages and promoting a shift from the inflammatory M1 macrophage subtype to the anti-inflammatory M2 subtype [17]. Moreover, estrogen is involved in the stability of blood vessels and cell membrane structures [30], and its interaction with CrM may have led to the suppression of inflammatory cytokines and to a reduction in vascular permeability. This potential synergistic effect has also been suggested for combinations of estrogen with other nutrients. For example, it has been reported that the presence of both vitamin D and estrogen enhances protective effects on bone and metabolic health [31]. This finding supports the idea that the physiological effects of estrogen may be enhanced through interactions with specific nutrients, and reinforces the physiological validity of the synergistic effects with CrM observed in this study. However, one limitation of the present study is that we did not control for or record the menstrual cycle phase in female participants. Hormonal fluctuations during the menstrual cycle—particularly changes in estrogen and progesterone levels—have been shown to influence inflammation, vascular permeability, and muscle membrane stability, which may in turn affect recovery from EIMD. According to a recent review by Cabre et al. [32], although there is currently no consensus on whether female sex hormones directly enhance muscle recovery, estrogen has been suggested to exert anti-inflammatory and fascia-stabilizing effects that could theoretically modulate post-exercise recovery. Therefore, the absence of menstrual phase tracking in our study represents a potential confounding factor. Future studies should consider stratifying participants according to their menstrual cycle phase or controlling for hormonal fluctuations to better elucidate the interaction between CrM supplementation and female hormonal status. Furthermore, it is also important to interpret these findings with caution due to the large inter-individual variability observed in both the CRE and PLA groups. Although several group-level differences reached statistical significance, their physiological relevance may differ among individuals. Such variability could arise from differences in muscle fiber type composition, creatine transporter expression, baseline nutritional status, or genetic factors affecting creatine metabolism. Therefore, while CrM supplementation appears to promote recovery from EIMD at the group level, further research is needed to identify which subpopulations are most likely to benefit from it. This limitation should be taken into account when applying our findings to practical settings such as athletic training or clinical rehabilitation.

We also found results consistent with those of prior studies [6] targeting young males in their 20s when evaluating the effects of CrM supplementation across a broader age range. This indicates that CrM has the potential to promote muscle function recovery regardless of age. According to previous studies, CrM supplementation is effective in improving muscle strength in adults between 30 and 50 years of age [33]. A recent systematic review and meta-analysis of randomized controlled trials reported that combining CrM supplementation with resistance training significantly improved upper and lower body strength, particularly in men [33]. The primary mechanism of CrM’s action lies in promoting adenosine triphosphate regeneration through the phosphocreatine system, which is considered to be less affected by age-related changes. Thus, the muscle recovery support provided by CrM is likely consistent, irrespective of age-related physiological changes. These findings support the notion that CrM supplementation is effective across a wide age range and can serve as a practical and accessible intervention for muscle recovery and performance enhancement. In this study, the average age of the participants was 26.1 years. Several individuals in their late 30s and early 40s were included. This allowed for some evaluation of age-related responses beyond early adulthood. However, it remains true that the absence of participants aged 50 years or older limits our ability to draw conclusions about CrM efficacy in older populations. Future research should aim to include a broader age spectrum, particularly middle-aged and elderly individuals, to better assess how aging influences muscle recovery and the effectiveness of CrM supplementation.

A limitation of this study is that dietary habits were not strictly controlled. CrM absorption and storage in the body are significantly influenced by dietary content, including carbohydrate intake and energy balance. For instance, Green et al. [34] demonstrated that the concurrent intake of CrM with carbohydrates significantly increases CrM accumulation in muscles through an insulin-mediated mechanism. Specifically, the concurrent intake of CrM and carbohydrates increased total CrM content in muscle by 60% compared to CrM intake alone [34]. Higher insulin levels enhance creatine transporter activity, promoting more efficient intracellular creatine uptake. Therefore, variations in the dietary patterns of the participants—including carbohydrate availability, meal timing relative to supplementation, and overall energy balance—may have contributed to individual differences in CrM efficacy. Stricter dietary control is necessary to evaluate the pure effects of CrM. In addition, hormonal factors such as menstrual cycle phase may also influence the recovery process, as discussed in more detail earlier in the manuscript. Future studies should incorporate hormonal status tracking to improve the interpretation of sex-specific responses.

This study provides new insights into how short-term CrM supplementation promotes recovery from EIMD and elucidates the potential role of CrM from multiple perspectives, including sex-based differences, muscle membrane stabilization, and muscle stiffness. The findings suggest that CrM supplementation promotes recovery from EIMD and has potential applications in both competitive sports and rehabilitation. From a practical perspective, the accelerated recovery of MVC by approximately 18.5% at 48 h post-exercise in the CRE group compared to the PLA group may allow athletes to resume high-intensity training earlier, reducing downtime and maintaining performance levels. In sports, shortening recovery time between games and training sessions can help maintain performance and increase training frequency. Similarly, the observed reduction in muscle fatigue by up to 25% at its peak highlights the utility of CrM in rehabilitation contexts, where rapid functional restoration is critical. In rehabilitation, CrM’s anti-inflammatory and membrane-stabilizing effects may promote postoperative or post-traumatic recovery and prevent joint ROM decline. For older individuals and women in particular, hormonal interactions may lead to a more pronounced suppression of edema and inflammation. Future research should focus on participants’ training history, dietary content, and age-related changes in muscle characteristics to optimize recovery strategies using CrM. This will contribute to the establishment of practical and individualized EIMD recovery approaches in sports and rehabilitation settings.

## 5. Conclusions

In this study, we investigated the effects of CrM supplementation on recovery from EIMD in a population comprising different sexes and age groups. The results showed that the CRE group, after 33 days of CrM intake, exhibited enhanced recovery of MVC compared to the PLA group. Moreover, reductions in shear modulus and perceived muscle fatigue were observed in the CRE group. Notably, in female participants, CrM supplementation tended to suppress the increase in ECW following exercise, suggesting a potential sex-specific effect of CrM. For example, MVC recovered approximately 18.5% more in the CRE group than in the PLA group at 48 h post-exercise, and muscle fatigue scores were reduced by up to 25%, indicating that CrM intake not only has statistically significant effects but also that these are meaningful in practical terms. These findings indicate that CrM may play a role beyond being a simple ergogenic aid, potentially accelerating muscle repair and contributing to the maintenance of muscle function after EIMD. CrM supplementation could serve as a beneficial nutritional strategy, not only for athletes but also for older adults and general fitness practitioners. In particular, the observed suppression of edema in female participants highlights the need for further research on the mechanisms of CrM, including its interaction with estrogen. Overall, the results of this study suggest that CrM supplementation may promote recovery from EIMD and be effective as part of strategies for maintaining sports performance and rehabilitation. Future studies should focus on optimizing CrM supplementation protocols that account for an individual’s body composition and hormonal status, as well as investigating the long-term effects of CrM intake on muscle function. Even though creatine supplementation may enhance recovery following exercise, potential effects on overall health should be taken into account. Therefore, its use should be supervised by a qualified healthcare professional.

## Figures and Tables

**Table 1:** Baseline variables measured prior to the 33-day supplementation period, physical characteristics, and indices of exercise-induced muscle damage.

			Levene		*t*-test		
	PLA	CRE	*F*-Value	*p*-Value	*t*-Value	*p*-Value	*t*-Test
Age (years)	26.8 ± 8.4	25.1 ± 7.1	0.566	0.456	0.865	0.392	n.s.
Body mass (kg)	60.0 ± 12.0	59.1 ± 8.6	1.866	0.180	0.417	0.679	n.s.
Body fat (%)	26.0 ± 6.4	23.0 ± 7.9	1.480	0.232	0.761	0.451	n.s.
SMM (kg)	27.0 ± 5.9	24.7 ± 5.3	0.061	0.806	1.217	0.231	n.s.
ROM (deg)	124.1 ± 7.0	123.8 ± 6.7	1.133	0.717	0.121	0.904	n.s.
MVC (kgf)	15.1 ± 5.0	15.2 ± 4.1	1.114	0.292	−0.057	0.955	n.s.
CIR (cm)	25.5 ± 3.4	25.4 ± 2.4	1.935	0.173	0.102	0.919	n.s.
SOR (mm)	0.0 ± 0.0	0.0 ± 0.0	−	−	−	−	n.s.
MF (mm)	0.0 ± 0.0	0.0 ± 0.0	−	−	−	−	n.s.

PLA: placebo group; CRE: creatine group; SMM: skeletal muscle mass; ROM: range of motion; MVC: maximum voluntary contraction; CIR: circumference, SOR: soreness; MF, muscle fatigue; n.s., not significant.

## Data Availability

The original contributions presented in the study are included in the article; further inquiries can be directed to the corresponding author.

## Supplementary Materials

The following supporting information can be downloaded at: https://www.mdpi.com/article/10.3390/nu17111772/s1, Table S1: Creatine_EIMD_Recovery_Dataset.

## Abbreviations

The following abbreviations are used in this manuscript:

CrM	Creatine monohydrate
EIMD	Exercise-induced muscle damage
MVC	Maximal voluntary contraction
ROM	Range of motion
CRE	Creatine supplementation group
PLA	Placebo group
BB	Biceps brachii muscle
Br	Brachialis muscle
SDs	Standard deviations
Xc	Reactance
PhA	Phase angle
TBW	Total body water
ECW	Extracellular water
ICW	Intracellular water
